# “Just-in-time” generation of datasets by considering structured representations of given consent for GDPR compliance

**DOI:** 10.1007/s10115-020-01468-x

**Published:** 2020-04-15

**Authors:** Christophe Debruyne, Harshvardhan J. Pandit, Dave Lewis, Declan O’Sullivan

**Affiliations:** ADAPT Centre, Trinity College Dublin, Dublin 2, Ireland

**Keywords:** GDPR, Consent, Data integration

## Abstract

Data processing is increasingly becoming the subject of various policies and regulations, such as the European General Data Protection Regulation (GDPR) that came into effect in May 2018. One important aspect of GDPR is informed consent, which captures one’s permission for using one’s personal information for specific data processing purposes. Organizations must demonstrate that they comply with these policies. The fines that come with non-compliance are of such importance that it has driven research in facilitating compliance verification. The state-of-the-art primarily focuses on, for instance, the analysis of prescriptive models and posthoc analysis on logs to check whether data processing is compliant to GDPR. We argue that GDPR compliance can be facilitated by ensuring datasets used in processing activities are compliant with consent from the very start. The problem addressed in this paper is how we can generate datasets that comply with given consent “just-in-time”. We propose RDF and OWL ontologies to represent the consent that an organization has collected and its relationship with data processing purposes. We use this ontology to annotate schemas, allowing us to generate declarative mappings that transform (relational) data into RDF driven by the annotations. We furthermore demonstrate how we can create compliant datasets by altering the results of the mapping. The use of RDF and OWL allows us to implement the entire process in a declarative manner using SPARQL. We have integrated all components in a service that furthermore captures provenance information for each step, further contributing to the transparency that is needed towards facilitating compliance verification. We demonstrate the approach with a synthetic dataset simulating users (re-)giving, withdrawing, and rejecting their consent on data processing purposes of systems. In summary, it is argued that the approach facilitates transparency and compliance verification from the start, reducing the need for posthoc compliance analysis common in the state-of-the-art.

## Introduction

As noted in [1]: *“computing professionals involved in “big data” research should pay attention if they wish to gain access to datasets containing or derived from personal information”.* While the article was written for data science researchers, this statement is true for all data processing purposes. Data processing, in general, is increasingly the subject of regulations, such as the European General Data Protection Regulation^1^ (GDPR). Such initiatives spur the investment in means and resources that will, in novel ways, facilitate compliance verification [2]. These initiatives are motivated by the fines that come with being non-compliant, such as up to 4% of an organization’s global revenue for GDPR.

Semantic and Linked Data technologies have proven that they can facilitate interoperability, transparency, and traceability in organizations [3]. This is why these technologies are being adopted to facilitate GDPR compliance verification processes. Our prior [2, 4–6] and related [7, 8] work addressed problems of representing aspects of GDPR [2, 4, 6], the various interactions between stakeholders [2, 6] and representing (informed) consent [2, 5, 9]. Only a few studies, including ours, demonstrate how this information can be rendered actionable, namely: [5] maps consent information to eXtensible Access Control Markup Language (XACML) for managing access to data; and SPECIAL [8] analyses the compliance of a process before execution. Rather than analysing the processes (before or after execution), we want to investigate whether an organization can also use this information for the creation of a compliant dataset, therefore, guaranteeing a compliant execution of a process. This would be useful for data processing activities (e.g. sending newsletters, using one’s data and history for a recommender system, etc.) that require one’s informed consent. This is challenging as one can give and withdraw their consent for (certain) data processing purposes at any given time, and the information used for these data processing purposes is not necessarily centralized. The problem we address in this article is thus how to generate datasets “just-in-time” (JIT) that are compliant with certain regulations such as the consent information required by GDPR.

There are reasons to use the Resource Description Framework (RDF). First, organizations often hold data in various heterogeneous data sources, motivating the need for a common graph-based data model, and for standardized technologies for transforming non-RDF data in RDF (called “uplift”). Note also that transforming RDF into non-RDF (called “downlift”) is often straightforward (e.g. [10] for RDF to CSV, and [11] for RDF to XML). Secondly, with RDF and its surrounding technologies (query languages, reasoning capabilities, provenance models, etc.) being already standardized and well established, one can easily avail of existing tooling. Python, R, and Java are all popular programming languages with mature libraries for processing RDF—with Python and R being popular for data analysis.

We generalize, extend, and integrate ideas previously reported in [9, 12]. Both studies looked into an RDF representation of multi-dimensional statistical datasets using the RDF Data Cube Vocabulary [13]. Debruyne et al. [12] presented a method for generating R2RML mappings that generate such datasets using Data Structure Definitions (DSDs). In [9], we demonstrated the feasibility of generating RDF Data Cube datasets that consider the consent an organization has gathered. In this article, we:

- Integrate both approaches;
- Introduce support for representing tabular data [11], thereby demonstrating its genericity; and
- Adopt and extend the Data Privacy Vocabulary (DPV) [14] vocabulary rather than our bespoke ontologies to integrate the various data sources.

This paper demonstrates how one can use Semantic Web technologies (which are open and standardized) to generate compliant datasets from a knowledge base of gathered consent information in a declarative manner. In other words, we used Semantic Web technologies to solve a problem in a specific problem domain; regulatory challenges in data processes. The remainder of this article is organized as follows. We formulate the objectives of this study in Sect. 2. In Sect. 3, we present the design of our solution and briefly elaborate on the various components: representing consent and consent information, generating mappings from annotated schemas, and the generation of datasets for GDPR compliance—which are knowledge engineering activities. Those three components are elaborated in more detail in Sects. 4, 5, and 6, respectively. To demonstrate our approach, we avail of a running example throughout these three sections. We describe, in Sect. 7, the motivation for supporting tabular data next to RDF Data Cubes, and elaborate on the differences between the approach for tabular data introduced in this paper, and the approach for RDF Data Cubes introduced in [9]. Section 8 presents the service that we developed. We conclude the paper in Sect. 9.

## Objectives, scope, and assumptions

The objective of this study is to propose a method for generating datasets in a “GDPR-aware” manner, with a focus on consent and fit for particular data processing purposes. We hypothesize that such an approach would facilitate compliance verification, facilitate transparency, and reduce the need for posthoc compliance analysis. We break down this objective into the following specific objectives:

1. To design and develop a model for representing the consent gathered by an organization and its relations to a system’s data processing purposes.
2. To propose a method for annotating schemas for the generation of compliant datasets “just-in-time”.
3. To develop the various processes in our approach in a declarative manner, adopting standardized vocabularies and techniques to maximize transparency.
4. To integrate the various components in a proof-of-concept service for the demonstration and evaluation of our approach.

We assume the consent (and the information thereof) an organization has stored is valid. In other words, we assume that the consent collected and stored satisfies all the requirements for valid consent under GDPR.^2^ How that consent was obtained is outside the scope of this paper. We also note that we are primarily concerned with the generation of datasets for data processing purposes. Compliance verification of activities when data is to be shared with third parties is not within the scope of this article.

## Design

**Fig. 1:**
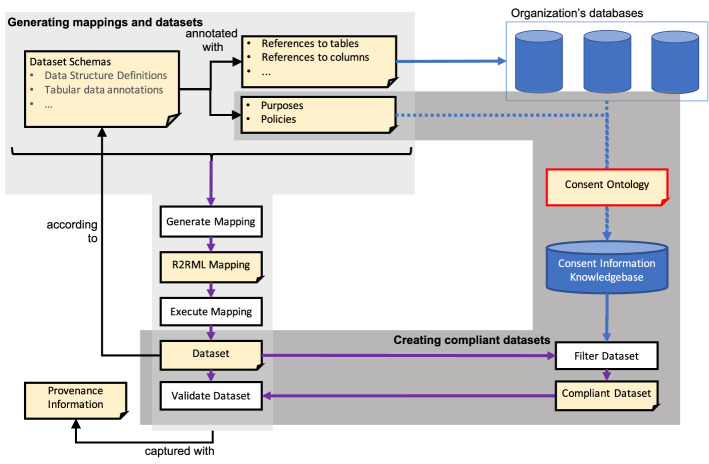
Overview of artefacts and processes in our architecture. Dog-eared rectangles represent artefacts and rectangles represent processes. The consent ontology (an artefact) is used to populate a consent information knowledge base from an organization’s databases (not necessarily relational)

We decompose the design and development of our solution in different components: an ontology for representing consent, the generation of *uplift*^3^ mappings, and the component that uses these two for the generation of compliant datasets. In GDPR, the term “purpose” refers to “the aim or goal towards which the data is processed (or associated with any other form of action)” [6]. Figure 1 depicts the various processes (rectangles) and artefacts (dog-eared rectangles) in our architecture. The figure furthermore depicts an organization’s databases that are involved as well as a knowledge base containing consent information.

- The **consent ontology**, shown in Fig. 1 as an artefact with a red outline, is presented in Sect. 4. We stated in the introduction that prior work relied on bespoke ontologies that we had developed. In this article, we have adopted the Data Privacy Vocabulary (DPV) [14], which is an initiative by the W3C Data Privacy Vocabularies and Controls Community Group to specify an ontology of terms for annotating personal data handling in the context of GDPR. As the community aims to standardize this vocabulary, we expect our proposed solution will have greater uptake by adopting and, extending this ontology as needed. Our approach extends the ontology by integrating the provenance ontology PROV-O [15] for the evolution of consent and with additional predicates for annotating and interlinking data schemas with DPV (see later). Our consent ontology is used to annotate a dataset schema description with a data processing purpose as well as to populate a knowledge base with previously gathered consent. That knowledge base will be a key in the “just-in-time” creation of a GDPR-compliant dataset (shown in dark grey).
- In light grey, we show the processes and artefacts involved in the **generation of uplift mappings** and the execution of those mappings to generate datasets. To generate those by the mapping engine, we adopt vocabularies to represent datasets and annotate those with references to tables, columns, and so on. The mapping engine will process both the schema and the annotations to generate an R2RML [16] mapping, a W3C Recommendation for declaring how relational data should be transformed into RDF. The R2RML mapping is then executed by an R2RML processor, producing a dataset. The datasets will, itself, be validated against the dataset’s schema. We elaborate on this process in Sect. 5.
- In dark grey, we show the processes, artefacts, and consent knowledge base involved in the **creation of a compliant dataset** process. This component interrogates the previously generated dataset and the consent knowledge base to filter out all information that should not be included in the dataset. We motivate filtering the dataset after the execution of the mapping in Sect. 6.

We will cover related work from the state-of-the-art related to each component in each respective subsection. The service that integrates all the various processes is presented in Sect. 8.

We stated in the introduction that prior work, presented in [9, 12], looked into the generation of R2RML from annotated Data Structure Definitions (DSDs) and the generation of compliant datasets from these R2RML mappings. A DSD can be regarded as a schema for (statistical) datasets, and a DSD can be described with the RDF Data Cube Vocabulary [13] specification. The use of this vocabulary for representing datasets may, at times, become fairly complex for “simple” tabular or relational data. For this reason, we have extended our approach to supporting tabular data represented as RDF using a model for tabular data on the Web—CSVW [11]. In Sect. 7, we discuss the challenges of using the RDF Data Cube vocabulary in our approach. The adoption of tabular data not only simplified the data that one can use for data processing, but also reduced the number of steps for generating an R2RML mapping. Section 8 will, thus, also present the differences in the generation of R2RML and compliant datasets. One of the key differences between RDF Data Cube and CSVW is expressiveness. In RDF Data Cube, one needs to explicitly indicate which values identify “records” and it includes the predicates for doing so. In CSVW, it is not mandatory to declare a schema in which you indicate the columns identifying a record. We thus need to provide a way to declare such columns in our approach. We will prescribe the use of dct:identifier as a special predicate for indicating these columns, which will be explained later on.

## Representing consent information

In November 2019, the W3C Data Privacy Vocabularies and Controls Community Group published the first version of their Data Privacy Vocabulary [14] or DPV. DPV is used to describe personal data handling. The ontology we developed for prior work (in [9]) was built from the perspective of an organization and only concerned with whether consent was given by data subjects. While our prior work [9] had most of the concepts and relations we need for our study, our proposed solution will likely have more impact by adopting the appropriate concepts and relations from DPV instead, given that the W3C community group aims to standardize the vocabulary. The concepts and relations we needed to include in our consent ontology are mostly related to the appropriate DPV ones and also linking dataset schemas with an organization’s data.

**Fig. 2:**
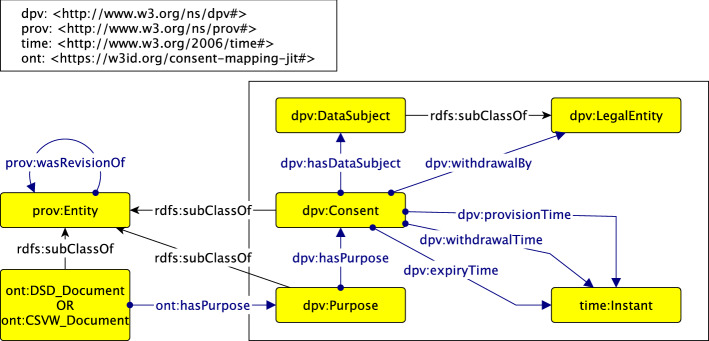
Main concepts and interrelations of our model

The main concepts and interrelations of the model are shown in Fig. 2 (modelled with Graffoo [17]). We note that both the domain of dpv:hasPurpose and the range of dpv:hasDataSubject is the union of dpv:Consent and dpv:PersonalDataHandling. For this article, however, we are only concerned with dpv:Consent and have omitted the concept-disjunction^4^*from the Figure*, for the reader. To further simplify the Figure for the reader, we also depict the range of provision-, withdrawal-, and expiry times to be time:Instant instead of its superclass time:TemporalEntity. While the time ontology also provides constructs to model durations, we only use date and time instances in this study.

Although DPV provides most of the classes and relations we need (i.e. around Data Subject, Consent, and Purpose), we needed to introduce the link between a dataset (schema) and a data processing purpose.

In DPV, the class “Consent” represents the consent given by a Data Subject (in most cases) or their legal representative. In this, each consent is considered unique for a given combination of Data Subject, Purposes, Processing operations, and Personal Data. DPV provides attributes to model how and when the consent that has been given and subsequently withdrawn. In our scenario, we also wish to model the situation where a Data Subject chooses to not give their consent, which is something that DPV cannot currently represent. Additionally, the DPV also does not provide information regarding modelling of information regarding the evolution of consent over time—such as a Data Subject providing consent that has been expired—was previously withdrawn, or was refused. To model this information, we introduced PROV-O [15] into our consent ontology which allowed us to capture revisions of consent in the form of provenance of entities. DPV provides constructs for annotating consent instances with the dates and times they were given (dpv:provisionTime) and the time they were withdrawn (dpv:withdrawalTime). Temporal dimensions in DPV are captured using the Time Ontology.^5^ We also avail of dpv:expiryTime to capture the date on which a data subject’s consent expires. The class “Purpose” is used to represent the purpose of processing (personal) data, which may range from using email addresses to send a newsletter to using one’s profile and purchase history for targeted advertising.

DPV only aims to provide a vocabulary and therefore does not prescribe methods for detecting erroneous or incomplete consent information. Rather than availing of Web Ontology Language (OWL) reasoning and the challenges that come with the Open World assumption, we validate our knowledge base with SHACL (Shapes Constraint Language) [18] which is the W3C Recommendation for prescribing conditions called ‘shapes’ which valid RDF graphs in a knowledge base must adhere to. SHACL is especially useful for closed-world assumptions and cardinality constraints which are the key constraints in our conceptual model. To summarize, we check whether there are no issues in the knowledge base at the graph level (i.e. RDF data model) rather than the semantic level (i.e. OWL). Another advantage of SHACL is the generation of machine-readable test results that can be utilized for documentation and compliance purposes.

### Consent ontology: related and prior work

The work presented in [2] is, in short, focused on adopting Semantic Web technologies and extending standardized vocabularies for (1) prescribing the actions that should take place as well as the interactions between various stakeholders in the context of GDPR and (2) analyse these models with respect to annotated logs and questionnaires to facilitate compliance verification. The work presented in [2] is from a prescriptive level concerned with the datasets that data processing activities use, who and how consent was obtained, and whether data processing activities comply with the consent that is and will be collected. While there is an overlap in terminology and the application domain, the model we propose in this article is meant to allow agents to utilize consent information available to an organization for assisting in the processing based on that consent and is not concerned with the assessment of the validity of consent for compliance. The model we propose in this article is intended to assist an organization in following the obligations of GDPR by generating a dataset taking into account the permission provided by given consent. Though there are some nuances in the interpretation of some terminology used in both studies, they are complementary in a broader narrative.

SPIRIT [7], SPECIAL [8], and the work presented in [19] have concepts related to data subjects, purposes, and data processing activities that are relevant. Those vocabularies were, however, mostly developed for a posteriori compliance analysis of logs or assessing operations before their execution. In SPECIAL, their vocabulary is furthermore used to analyse whether a data controller’s policy complies with a data subject’s consent [20]. The SPECIAL compliance checker is used at an individual level for every data subject’s consent. Our goal is to produce an entire dataset for a given collection of consent. The SPECIAL compliance checker also uses OWL2 subsumptions to dynamically check whether a given purpose or processing is compatible with a given consent which, while being more flexible, is rarely a dynamically changing operation as organizations have purposes that do not change frequently. While the intended uses of these vocabularies are different, their integration with the vocabularies we have introduced in this section will be challenging due to semantic heterogeneity of terms involved. An alignment of these, which would result in ontology mappings facilitating interoperability, is considered for future work, though we note that the core vocabulary developed in the SPECIAL project informed DPV’s base vocabulary.

Most relevant to the Consent Ontology is GConsent, which we proposed in [6]. GConsent recognizes that most initiatives do not consider all aspects of consent in order to appropriately assess whether consent fulfils the requirements provided by GDPR. GConsent therefore aims to capture the “context” of consent along with its evolution as an entity based on the notion of distinct states such as not given to given to withdrawn. GConsent represents verbose information about consent from the perspective of compliance requirements rather than an organization’s perspective. Consent Receipt [21] is a standard by the non-profit Kantara Initiative that specifies attributes for capturing a consent record in the form of the human-readable receipts primarily intended for data subjects. The current iteration of Consent Receipt (v1.1) is based on the ISO/IEC 29100:2011 terminology, which is different from that used by the GDPR.

## Generating uplift mappings in R2RML

To exemplify our approach, we will introduce a running example.^6^ In our example, we simulate an organization who aims to send a newsletter to their customers (a data processing purpose). Newsletters can only be sent to customers who have given their explicit consent. In our example, we have a relational database table “Customer” that contains last names, first names, and email addresses (amongst other things) (Table 1).

**Table 1:** The customers table

id	email	first_name	last_name	…
1	user_1@example.org	Firstname 1	Lastname 1	
2	user_2@example.org	Firstname 2	Lastname 2	
3	user_3@example.org	Firstname 3	Lastname 3	
…	…	…	…	…

We will use that table to a dataset schema file referencing that information. We assume, for the running example, that the dataset schema file will be stored at a particular location,^7^ this will be important as the location of the file (an absolute URI) will be the base of the RDF graph. When creating a dataset for data processing purposes, a location (and thus, also, an absolute URI) will be generated

### Step 1: annotating the schema

We start off with the (re)use of a schema describing the tabular data, which we will annotate with information on where to fetch the data. Listing 1 depicts an RDF graph containing a minimal dataset definition of the tabular data using the CSVW vocabulary and JSON-LD representation provided by [10]. We only retained the name of the columns as well as the property URLs giving us an indication as to how values in these columns must be interpreted. The vocabulary allows one to prescribe mandatory constraints (i.e. are values required), data types, and patterns that values should comply with (amongst others). These constraints are useful for validating tabular data. The highlighted statements extend the tabular file definitions with mapping information. We refer to a relational database table called “Customer” with the structure depicted in Table 2.

**Table 2:** Schema of the relational database table “Customers”

Column	Type	Null	Key
id	bigint(20)	No	Primary
first_name	varchar(50)	No	
last_name	varchar(50)	No	
email	varchar(255)	No	Unique
…	…	…	…


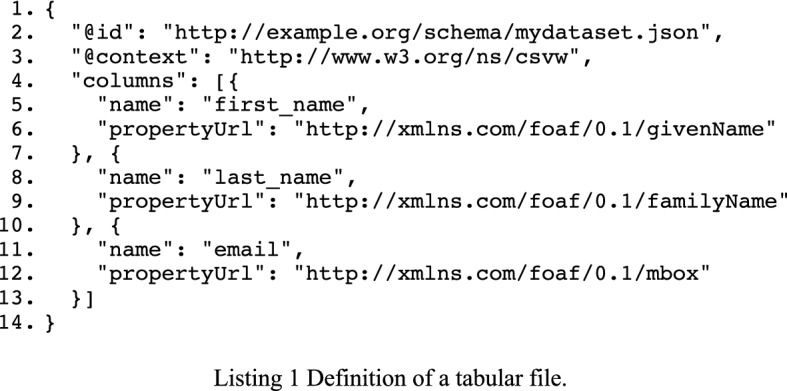


We reuse R2RML’s predicates to further annotate the dataset schema, as R2RML already provides us with the necessary predicates to annotate the file’s schema. Those predicates are used to indicate where one can find the source tabular information in a relational database (see Listing 2, highlighted). It is important to note that we do not intend to create a valid R2RML document by reusing those predicates. We will, however, use them to generate a valid R2RML mapping in the next step of the process.


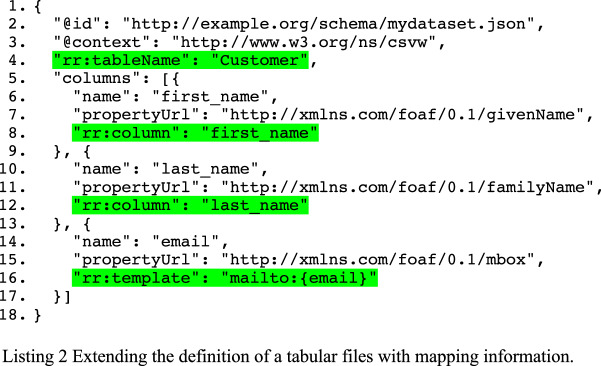


The dataset schema is also serialized in JSON-LD [22], a common and increasingly popular representation for RDF for the available tooling. One can translate this JSON-LD into another RDF serialization such as TURTLE, but most of the column definitions will have no identifier (i.e. they will be blank nodes). Our approach of using RDF Data Cube had the additional benefit of having identifiers (URIs) for the different parts of the schema. This allows for an easier separation between schema and annotations. While this is also possible in JSON-LD, the way RDF Data Cube structures the data “forces” one to provide identifiers for those various components.

### Step 2: generation of an R2RML mapping

We have chosen to adopt a declarative approach to generating the R2RML mapping via a sequence of SPARQL CONSTRUCT queries. The various queries can be summarized as follows: (1) create the triples maps (for mapping tables, views or queries to RDF); (2) use the columns to create subject maps; (3) create predicate object maps for the columns, and (4) connect the dataset that will be generated with its schema.

We obtain an executable R2RML mapping by merging the models resulting from each SPARQL CONSTRUCT query. This model is not meant to be merged with the prior RDF graphs from Listing 2. Instead, it will be used to generate RDF that will be the “just-in-time” dataset. In a wider governance narrative, the resulting mapping may be stored to inform stakeholders of the provenance of the datasets. We now begin with the description of each query. Note that we have omitted prefixes and base declarations from each of the query listings for brevity.

The generation of a logical table (tables and views) for each schema related to a table is the first query and is shown in Listing 3. A similar CONSTRUCT query is used for schemas related to a query with the rr:query predicate. The namespace ont: refers to our vocabulary developed for this study, and is useful in order to attach the different components of the R2RML mapping later on.


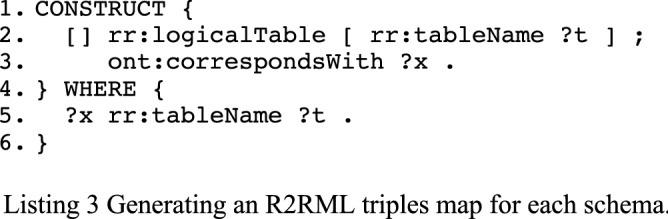


The CONSTRUCT query for generating the subject map is shown in Listing 4. The columns of the tabular data are used to identify each record. We use that information to generate the subject map of a triples map. The table columns used by the columns are used for creating a template that will identify each record in the dataset. An R2RML processor will use the template, which will generate values, to keep information of each record in an appropriate data structure (e.g. a dictionary). Notice on line 17, we rely on strSplit function offered by Apache Jena’s SPARQL processor^8^ to split a string into a list. Such a function was, unfortunately, not available in SPARQL. On lines 20 and 21 (grey), we include function calls which are part of an R2RML-F, an extension of R2RML [23].


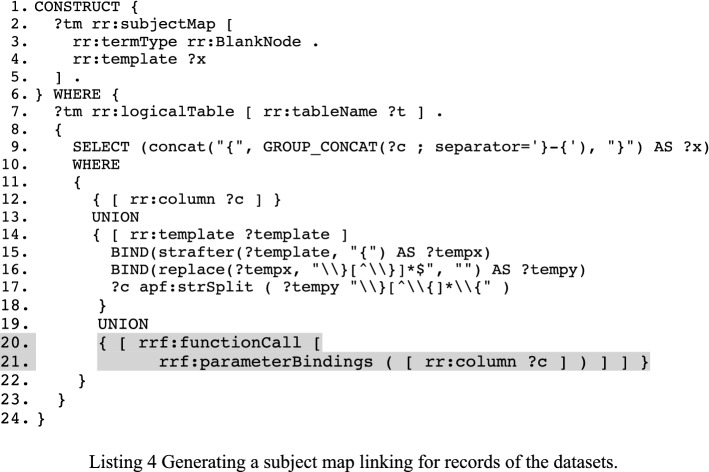


Listing 5 provides the CONSTRUCT query for adding predicate object maps to the triples maps based on columns. The query for tabular data is fairly straightforward. We test for the presence of a csvw:propertyURL that refers to an RDF predicate. As the use of csvw:propertyURL is not mandatory, we will construct a predicate based on the column’s name from the schema when that property is missing (lines 17–19). We note that a base URI—usually the URL or IRI of the dataset—is needed to provide absolute URIs of the column names. We avail of URI encoding to ensure that the URI of each predicate is valid.


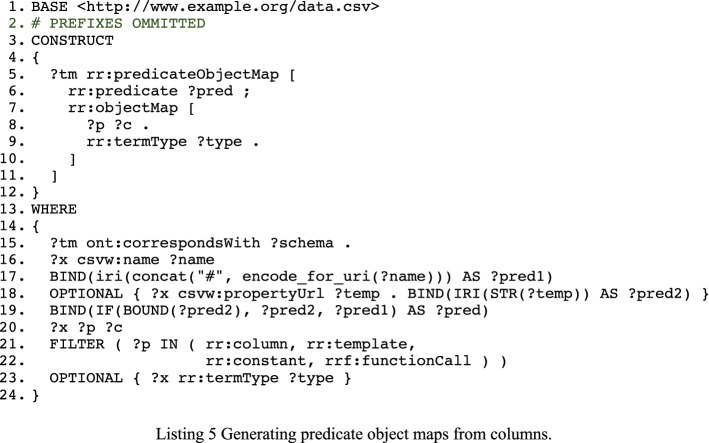


Finally, the dataset that will be generated with this dataset also needs to be connected to its schema. This is straightforward with the following CONSTRUCT query (in Listing 6). In CSVW, the schema refers to a particular file with the csvw:url predicate. We add this statement in the dataset that we will generate by executing the R2RML mapping as one of the final steps.


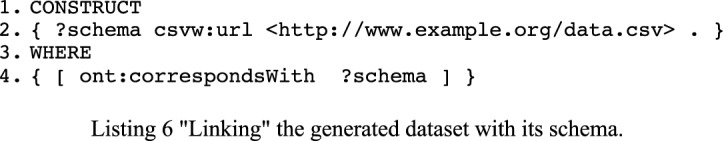


With these mappings—which are declarative and implemented as SPARQL CONSTRUCT queries—we are able to generate an executable R2RML mapping that will be used in the generation of the “just-in-time” compliant dataset in the next process. Given our table “Customer” in Table 2 and the snippets from Listing 2, the R2RML in Listing 7 is generated. While it is not explicit that the resource is a rr:TriplesMap, it will be inferred by the R2RML engine as such since the domain of rr:logicalTable is rr:TriplesMap.


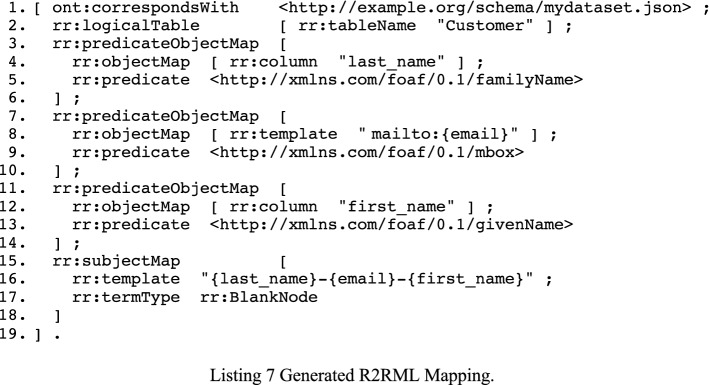


### Step 3: execution of the R2RML mapping

For the execution of our mapping, we rely on an implementation of the R2RML implementation developed by [23] as some use cases are not supported by the R2RML specification (see discussions). The mapping in Listing 7 contains no statements that fall outside R2RML’s scope and should work with other implementations of the specification. The execution of this mapping generated 3 RDF triples for each record in the Customers table, which corresponds with the generated dataset in Fig. 1. An example of such a record is shown in Listing 8, representing “user_1” in the graph with their first and last name.


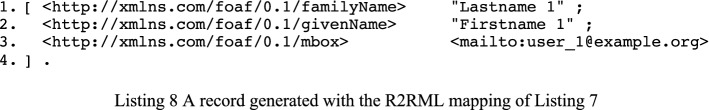


In the case of an XSD datatype, our R2RML processor checks whether a value that is generated by an object map corresponds with that datatype and reports when this is not the case. When a datatype is not part of the XSD namespace is used for an object map, such as ex:myInteger, for instance, the literal is merely typed with that datatype. If no datatype is provided, the datatype of the literal depends on the datatype of the column (see Sect. 10.2 “Natural Mapping of SQL Values” of [16].

### Step 4: validating the generated RDF

We validate the generated RDF by checking the integrity constraints described in the schema. This is necessary as the execution of any R2RML mapping according to a particular schema or standard does not guarantee that the resulting dataset complies with the constraints of that schema or standard. For RDF Data Cubes, the specification presents a set of so-called integrity constraints in the specification [13], which are a sequence of SPARQL ASK queries. For CSVW, we rely on CSVW validators^9^ taking as input both the schema and the generated dataset to check whether the dataset adheres to the schema’s constraint.

### Related work on generating mappings

Though tools exist to convert between representations (such as from OLAP and CSV to RDF Data Cube [24]), we are interested in the state-of-the-art of generating mappings from one representation to another. Related work in generating (R2RML) mappings from other representations is quite limited. The authors in [25]—who proposed a declarative language for ontology-based data access where a single description results in an ontology, mappings, and rules for transforming queries—mentioned adopting R2RML because of its increasing uptake.

The Open Cube Toolkit [26] provides a D2RQ [27] extension for generating an RDF graph according to the RDF Data Cube Vocabulary using D2RQ’s R2RML support. The D2RQ data provider requires a mapping relating a table to a dataset using a bespoke XML mapping language. The XML file is then used to generate an R2RML mapping which is then executed by D2RQ’s engine. Their approach is thus similar in that it generates an executable R2RML file from the mapping. The limitations of their approach relate to their mapping language; it is bespoke, not in RDF and has not been declared in a particular namespace.

## Creating compliant datasets

In this section, we will demonstrate our approach by (1) describing how we use a knowledge base using the ontology we presented in the previous section; (2) annotate the schemas for generating R2RML mappings (cf. Sect. 3.1); and (3) how we combine (1) and (2) to generate a consent-compliant dataset for use in data processing operations.

### Using the consent ontology

We retrieve consent information for a particular purpose using a SPARQL SELECT query (see Listing 9) upon the Consent Information Knowledge Base (whose information is structured according to the Consent Ontology). We ensure retrieving the most recent consent information for each user by removing those for which there is an instance of consent with a more recent date (lines 12–17). We then filter out those consent instances which have been withdrawn and are not expired (lines 19–24). Note, on line 19, that the consent can be withdrawn by a resource other than the data subject. While it is usually the data subject that gave their consent, there are cases where consent can be withdrawn (or given) by representatives (e.g. a parent for their child).


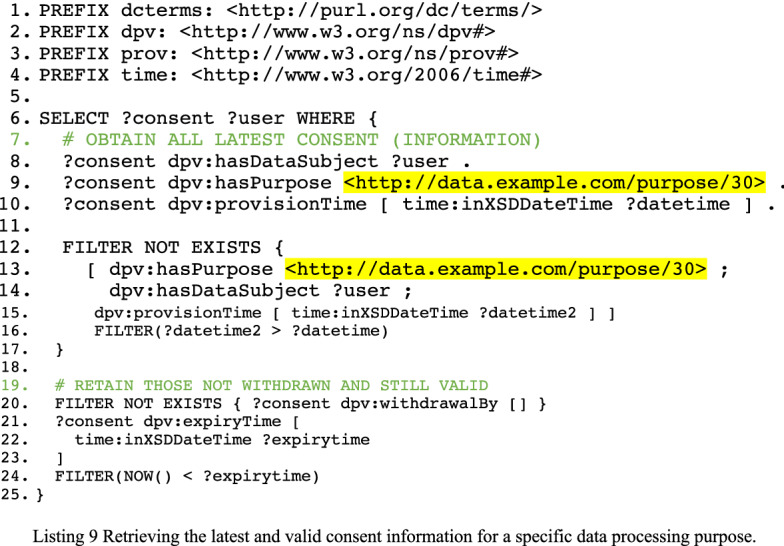


In Sect. 3, we annotated the schema with references to tables, columns, and so on. Similarly, we annotate the schema with references to a purpose. To exemplify this process, we will expand the running example for which we created a synthetic dataset. In that dataset, we have simulated ten users engaging with the terms and conditions of five systems. The terms and conditions of a system can evolve over time and, when they change, the consent has to be obtained again. Users can either reject, give, or withdraw their consent for a particular purpose at any given time. In this dataset,

- The policies of systems #4 and #5 were updated twice, and that of system #2 only once;
- There are 29 instances of users renewing consent;
- There are 68 instances of users re-giving consent after withdrawing; and
- Users withdrew their consent 102 times.

For the synthetic dataset, we have a simple schema (see Listing 10). We see in this listing that this schema retrieves data from a table and that the values for the dimension and measure are obtained from columns. Those values are used to create IRIs, one to identify records and one for mailboxes (according to the FOAF vocabulary). We note that the schema is annotated for a particular purpose (line 9)—an URI referring to sending newsletters. For the sake of brevity, however, we have omitted the first and last names from the annotated schema.


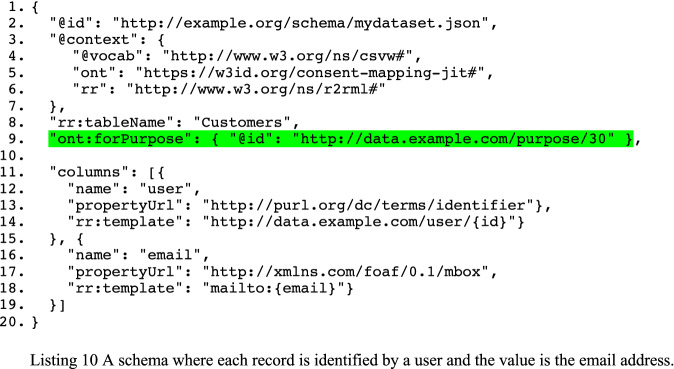


In RDF Data Cube, we must explicitly identify the components that identify an observation (corresponds with “records”). In CSVW, however, this is not mandatory. We, therefore, use dct:identifier for indicating which column should be interpreted as an identifier (line 13 in Listing 10). The reason being that CSVW prescribes the use of predicates whose node names reside in the base name of the file. For example, the column with name “name” will become the named node “name” in the file with URI http://example.org/schema/mydataset.json. The R2RML mapping that is the result of executing a sequence of the mapping generation CONSTRUCT queries is shown in Listing 11.


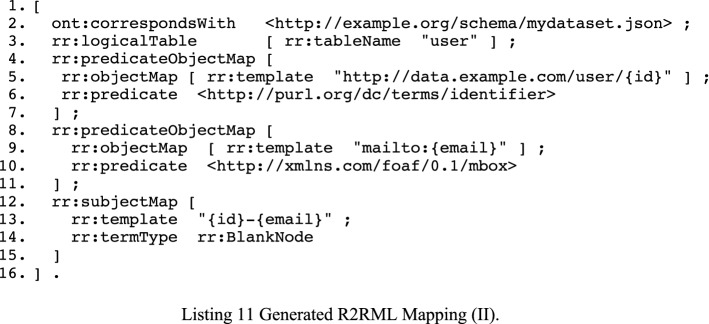


### Creating a compliant dataset

The synthetic dataset contains, for purpose #30 “sending newsletters”, consent information about three users. These users have given and withdrawn their consent several times in the 100 iterations. In the end, user #10 has accepted the purpose, whereas users #8 and #9 have withdrawn their consent. The execution of the R2RML mapping (Listing 11) results in a dataset that contains all users, but for our approach to work, we have to filter out only those for which we have explicit consent, in order to generate the target Compliant Dataset (as per Fig. 1). While we could manipulate the source of the R2RML mapping (by creating an SQL query with necessary WHERE clauses) to do the filtering, we choose to use SPARQL instead. This approach allows us not only to cache intermediate outputs but also allows us to avail of R2RML dialects that support non-relational data such as RML [28] and xR2RML [29]. In other words, we become less dependent on underlying database technologies that may differ across (different parts of) organizations and rely on RDF and SPARQL as the common model.

With the query in Listing 9, we fetch the latest consent information of users. This information is used to construct a list of users that have given their consent. That list is then used to create and execute a SPARQL DESCRIBE query returning to us a dataset that is fit for the purpose for which consent has been agreed. The list is used to create a VALUES clause. The DESCRIBE query is shown in Listing 12 and is applied to the dataset to obtain a subset with only the information of those that have given their consent.


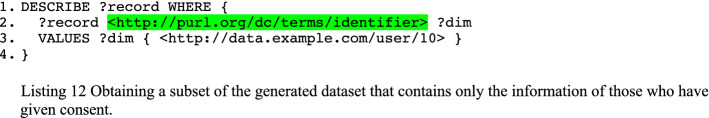


The predicate highlighted in Listing 12 corresponds with the predicate of the identifier used in Listing 10. We inspect the schema to obtain those when creating the DESCRIBE query. We omit this rather simple SPARQL query.

We already stated that for this particular purpose, user #10 has given their consent, and users #8 and #9 have withdrawn their consent. Because of this, only user #10 is retained in the result, Listing 13 shown below. If multiple records were retained, then the VALUES clause in Listing 12 would have contained multiple IRIs separated by spaces. This dataset is validated with the same techniques as Sect. 5.4.


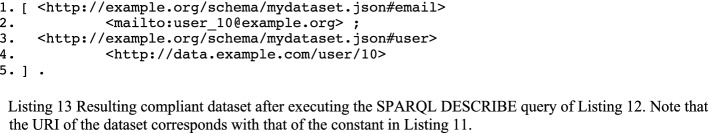


### Provenance information

Capturing provenance information is not a separate step, but rather a process that is happening throughout the steps of generating an R2RML mapping and compliant dataset. Provenance information provides insights on a resource’s origin, such as who created that resource, when it was modified, or how it was created [30]. PROV-O [15], which we already adopted for the consent ontology of this study, is a W3C Recommendation for representing and exchanging provenance information as RDF. PROV-O’s core concepts and relations provide a good starting point for describing the activities and intermediate artefacts that lead to the realization of an ontology mapping.

Rather than providing a snippet of the generated RDF, we will describe how we extended PROV-O and how the entities are used an interrelated. The classes we have declared in our namespace and which PROV-O concepts they specialize are shown in Table 3. Our proof-of-concept relies on R2RML-F, which will be an instance of ont:R2RML_Processor and ont:Mapping_Generator will be instantiated for our implementation.

**Table 3:** Extending PROV-O

Class	Parent class	Description
ont:DSD_Document ont:CSVW_Document	prov:Entity	Used to represent RDF graphs containing our annotated schemas
ont:R2RML_Mapping	prov:Entity	Used to represent the generated R2RML mappings
ont:Dataset	prov:Entity	Used to represent the generated datasets
ont:Compliant_Dataset	prov:Entity	Used to represent the final datasets
ont:Validation_Report	prov:Entity	Used to represent the validation reports
ont:Generate_Mapping	prov:Activity	Represents the activity of generating an R2RML mapping from an annotated schema
ont:Execute_Mapping	prov:Activity	Represents the activity of executing the R2RML mapping
ont:Filter_Dataset	prov:Activity	Represents the activity of filtering the dataset for creating a compliant dataset
ont:Validate_Dataset	prov:Activity	Represents the activity of validating a dataset
ont:Mapping_Generator	prov:SoftwareAgent	Represents the software agent that generates an R2RML mapping as per our approach
ont:R2RML_Processor	prov:SoftwareAgent	Represents the software agent executing the mapping
ont:Validator	prov:SoftwareAgent	Represents the software agent validating the dataset

We declare relations between instances of our subclasses to capture the whole process. For instance, a ont:Generate_Mapping uses (prov:uses) a ont:CSVW_Document to generate a ont:R2RML_Mapping. A mapping is thus generated by (prov:wasGeneratedBy) an activity. This activity was performed (prov:wasAssociatedWith) by our implementation of our approach (ont:Mapping_Generator). The mapping is also derived from the ont:CSVW_Document, so we also assert a prov:wasDerivedFrom between the two entities. We also store timestamps (start- and end-time) of each activity. The adoption of PROV-O in this study allows us to create traceable data flows where a schema can be used to generate an executable R2RML document multiple times. This helps us fulfil some of the requirements put forward by policies.

As for related work on creating compliant datasets, there is little relate work. To the best of our knowledge, relevant academic work on generating compliant datasets “just-in-time” (i.e. on-demand) is limited to [9].

## On generalizing the approach

In this section, we provide the motivation for supporting tabular representations next to RDF Data Cubes as well as the differences in the implementation (i.e. the SPARQL queries). This allows one to identify which parts of our approach need to be addressed if one were to provide support for another representation.

### Motivating the adoption of CSVW

Prior work [12] focused on the generation of RDF Data Cube Vocabulary [13] datasets. The Data Cube Vocabulary allows one to represent multi-dimensional datasets using RDF. While the vocabulary’s underlying model is indeed an ISO standard for representing statistical data and its metadata, the vocabulary is generic enough to represent even simple “relational” data. As it is capable of representing a wide variety of datasets and is, unlike other vocabularies, standardized, it was deemed suitable for our purpose. However, we realized that RDF Data Cubes can become too complex for certain data processing tasks.

**Fig. 3:**
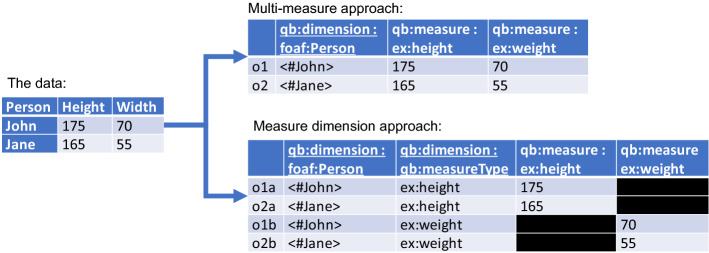
Left: height and width measured for two people. The measurement units of height and width were omitted but are assumed to be centimetres and kilograms. Right: the multi-measure approach for the example on the left and the measure dimension approach for the example for that same example. Note that we have omitted namespaces, but assume to be using URIs for instances of foaf:Person and predicates for weight and height from an arbitrary namespace. All predicates with the prefix qb reside in RDF Data Cube’s schema

The vocabulary works well when observations have at most one measured value. Problems may arise when one needs to capture multiple observations (e.g. rainfall and temperature, or width and height). The Recommendation proposes two approaches: a measure dimension approach and a multi-measure approach [13]. Take, for instance, the example on the left-hand side of Fig. 3. Here, we have a rather simplistic representation of two observations. The two observations o1 and o2 are identified by instances of people (dimension). For each person, we record their height and weight.

The multi-measure approach resembles the “relational” approach the closest but comes at an important price: expressiveness. With the Data Cube Vocabulary, one can only declare one unit of measurement per observation. Those can be declared as an attribute of the predicates, but then those are declared for the whole (and even all) datasets and you cannot use nits interchangeably (e.g. dbpedia:Centimetre and dbpedia:Metre).

In the measure dimension approach, observations are said to be identified by an additional dimension: the type of the measure. The Recommendation prescribes the use of qb:measureType to provide that additional dimension. The URI for the observed value does appear twice in each observation: once as the object for the qb:measureType property, and once as the predicate for the actual observed value. With this approach, units can now be attached for each observed value; e.g. dbpedia:Kilogram for weight and dbpedia:Centimetre for height. But one, then, has n observations per “row”, with n the number of measured values, creating a complex graph.

Users are thus faced with a rather difficult decision; limited “relational” data or expressive complex graphs. With these limitations in mind, we have chosen to adopt a tabular representation allowing for the annotation of tables and columns (datatype, cardinality, etc.) [10].

### Differences between RDF Data Cube Vocabulary and CSVW

This article has demonstrated how an initial approach to generating compliant datasets can be generalized to cater for different types of data; tabular data using an RDF representation of CSV files (which can be translated into CSV files according to the CSVW specification), and RDF Data Cubes based on the aforementioned prior work.

This article focuses on CSV files. Not only are these representations useful for a wider community (e.g. via the tooling available for processing JSON and CSV files), but also meant the instantiation of our approach was simpler as well.

Below we elaborate on the differences in terms of the “implementation” of the approach. Note that these are limited to the number of SPARQL CONSTRUCT queries that were required. In other words, the design of the approach is—from a conceptual perspective—the same, but RDF Data Cubes required additional transformations for the creation of a compliant dataset. The following are the key differences:

1. When dealing with RDF Data Cubes, we annotate the Data Structure Definitions. Those DSDs can be used for multiple datasets. When generating an R2RML mapping, we create a mapping for the generation of observations. Those observations have to be related to an instance of a dataset, which in turn is related to the DSD. When creating the subject map for RDF Data Cubes, we also: (1) include a predicate object map referring to an instance of a dataset (a constant), and (2) create a URI for that dataset based on the URI base.
2. For CSVW, each record is assumed to be identified by all the values. In RDF Data Cube, each observation is identified by its dimensions. The SPARQL CONSTRUCT query of Listing 4 will only consider column references used in dimensions.
3. The predicate object maps for CSVW are straightforward; a literal for each value. In RDF Data Cubes, there are dimensions and measures. We proposed a SPARQL CONSTRUCT query for each that resembles Listing 5. Key differences with respect to CSVW is that both can have optional term types (e.g. a resource or a literal) and ranges (e.g. XSD datatypes).
4. As for validating the datasets, RDF Data Cube prescribes a set of SPARQL ASK queries implementing so-called “integrity constraints” that test whether a dataset adheres to a Data Structure Definition. These SPARQL queries have been implemented in our service. For CSVW, the standard only prescribes what types of validations are supported by the specification. The community group behind this Recommendation also provided 282 test cases that validators should cover.^10^ Rather than implementing a validator, we availed of one realized with an MIT licence.^11^

We refer to [12] for more details on the implementation for RDF Data Cube.

## Design of the service

**Fig. 4:**
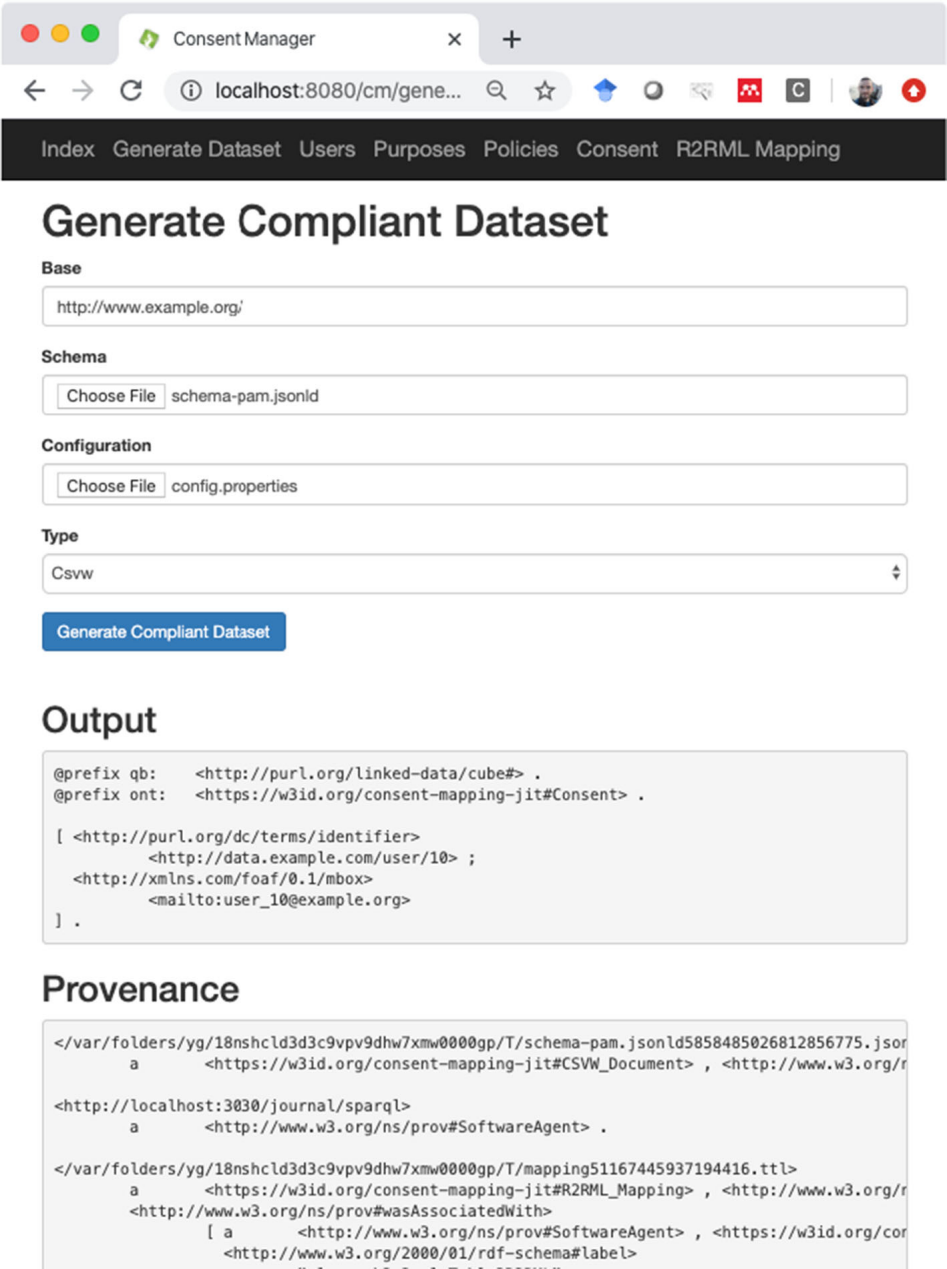
Interface built on top of the service. Given an annotated schema and details of the data sources (in a configuration file), the service generates a dataset taking into account the consent an organization has gathered as well as a provenance graph with details of each step as outlined in Sect. 3

We implemented our approach as a service. The service is implemented in Java EE and uses the Apache Jena^12^ to process the RDF. External libraries include R2RML-F^13^ as the R2RML processor and a CSVW validator which we referred to in the previous section. We also built an interface on top of that service (see Fig. 4) as a demonstrator, which was implemented with Apache Tapestry.^14^ First, we assume that one maps the consent information stored in some non-RDF format to our consent knowledge base (by using, for example, R2RML) and expose that information via a SPARQL endpoint. This assumption is motivated by the fact that organizations oftentimes store information in various (non-RDF) data sources. Both the service and the SPARQL endpoint are residing on a server and that the endpoint is not accessible from outside the system (i.e. behind a firewall). This is usually the case for any database in an organizational setting.

To use the service, one needs to input an annotated schema and a link to a purpose to obtain the filtered dataset for a particular data processing activity. In Fig. 4, the annotated schema includes links to a purpose in one file (see Listing 10). The tool also requires information about the non-RDF datastore (location or connection URL, username and password if needed, etc.). Where this configuration should be stored is not detailed by any of the W3C recommendations but depends on the various implementations. R2RML-F, the R2RML processor we have adopted, relies on a properties file. Our approach can be easily extended to store such information in separate graphs and instead point to the URI of a datastore description in RDF. We note that any governance activities related to creating the schema as well as the use of the obtained dataset are outside the scope of this paper.

Figure 4 furthermore depicts the results of running the service: we have the resulting dataset and the provenance graph. All (intermediate) files are to be stored in a secure location with references (URLs) in the provenance graph. This allows one to verify all files generated by the activities for posthoc compliance checking. Where and how these files should be stored—which is again concerned with governance strategies—falls outside the scope of this article. Although an appropriate manager should for these governance strategies, we currently store these files in RDF files in a separate folder.

**Fig. 5:**
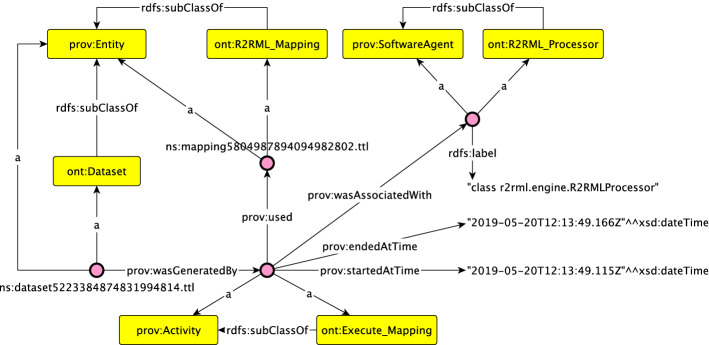
Visualization of some provenance information generated by the service. We have omitted namespaces and only display a part of the provenance graph for brevity

Figure 5 is generated using a tool called “ontology-visualization”,^15^ and depicts a part of the provenance graph generated by the tool. As the graph is quite large, we have omitted namespaces and only display the statements surrounding the activity of generating an R2RML mapping. This figure illustrates the detailed provenance information we can store. Again, the inclusion of additional information (such as the person executing the service) can easily be included but requires integration with data governance platforms.

## Conclusions and future work

We argue that datasets are used by an organization for a specific purpose and that datasets should be generated suitable for the intended purpose, including any organizational policies it should comply with. The question we addressed in this article is: “How can we generate datasets for a specific purpose “just-in-time” that complies with given consent?” We believe we answered this question by proposing a solution that:

1. Shows how we capture consent information by extending DPV, the Data Privacy Vocabulary which is on track for standardization. Since we had to relate dataset schemas with DPV, we had to include concepts for representing those schemas and the link between the schemas and dpv:Purpose. Our extension is used to access such information an organization has gathered for a specific purpose and for a specific schema. This corresponds with the first objective O1 of our research which we outlined in Sect. 2.
2. To achieve the second objective O2 “generating mappings”, we propose a method for generating R2RML mappings from annotated schemas. Those R2RML mappings, linked to a particular purpose and data source, are then used to generate RDF. Executing these mappings result in datasets that comply with the schema. This work was based on [12], and now generalized to cope with different types of datasets.
3. The third objective O3 “generating compliant datasets” proposed a declarative approach to manipulating the resulting datasets to exclude data for which no consent is given. This work was based on [9], and also generalized to cope with different types of datasets.
4. Finally, we achieved our fourth objective O4 by integrating all components into a service.

We have, thus, demonstrated that the use of Semantic Technologies for creating given consent-compliant datasets is not only feasible but also facilitates compliance verification processes and provides organizations with a compliance-by-design approach for their operations. These processes are facilitated by the declarative approach (i.e. queries), which is transparent. The generated mappings, datasets, and provenance data, in addition, can also be safely stored for posthoc analysis.

The system creates datasets with the goal of facilitating compliance. While it does so automatically, our approach allows one to look up why someone’s personal data was included in a particular dataset via the consent knowledge base, the sequence of SPARQL queries, and the timestamps captured in the provenance model.

In terms of future work, we identified in this article both the need for a fuller evaluation of the implemented system and also the need for alignment across existing consent related vocabularies.

We could not report as yet on the evaluation of our approach via quantitative methods or the demonstration of the approach in a testbed, as we have as yet no access to a real-world dataset of given consent. The state-of-the-art also provides little information on the actual data available and the complexity of the use cases. Instead, we relied on the demonstrator of Sect. 3 to convey the viability of our approach. We also created a script that generated synthetic data to support said demonstrator.

The challenge in aligning the various vocabularies is combining their respective scopes as well as their varying degrees of granularity (a specific type of heterogeneity). The work presented in this article, for instance, is meant to support data processing activities from an organizational perspective, whereas the work presented in GConsent [6] aims at representing the consent given from the perspective of the data subject. Where our model relates dataset schemas with consent, GConsent and DPV have more fine-grained representations for personal data and data categories. An attempt at aligning the vocabularies should take into consideration the scope and objective of two vocabularies and their respective roles in the compliance process? Such an alignment may, furthermore, have an impact on how people engage with the vocabularies (e.g. the complexity of queries).

## Footnotes

1 https://eur-lex.europa.eu/eli/reg/2016/679/oj.

2 https://www.privacy-regulation.eu/en/article-4-definitions-GDPR.htm, last accessed January 2020.

3 Uplift is the process of generating RDF from non-RDF resources [31].

4 ObjectPropertyRange(dpv:hasPurpose ObjectUnionOf(dpv:Consent dpv:PersonalDataHandeling)).

5 https://www.w3.org/TR/owl-time/, last accessed January 2020.

6 All files are available at https://www.doi.org/10.6084/m9.figshare.11925942.

7 E.g. http://www.example.org/data.csv.

8 https://jena.apache.org/, last accessed March 2019.

9 E.g., https://github.com/malyvoj3/csvw-validator.

10 https://w3c.github.io/csvw/tests/, last accessed January 2020.

11 https://github.com/malyvoj3/csvw-validator.

12 https://jena.apache.org/documentation/fuseki2/.

13 https://github.com/chrdebru/r2rml.

14 https://tapestry.apache.org/.

15 https://github.com/usc-isi-i2/ontology-visualization, last accessed May 20, 2019.
